# Using the Horse Grimace Scale (HGS) to Assess Pain Associated with Acute Laminitis in Horses (*Equus caballus*)

**DOI:** 10.3390/ani6080047

**Published:** 2016-08-03

**Authors:** Emanuela Dalla Costa, Diana Stucke, Francesca Dai, Michela Minero, Matthew C. Leach, Dirk Lebelt

**Affiliations:** 1 Department of Veterinary Medicine, University of Milan, Milan 20122, Italy; francesca.dai@unimi.it (F.D.); michela.minero@unimi.it (M.M.); 2 Havelland Equine Clinic, Beetzsee 14778, Germany; diana.stucke@pferdeklinik-havelland.de (D.S.); lebelt@pferdeklinik-havelland.de (D.L.); 3 School of Agriculture, Food & Rural Development, Newcastle University, Newcastle Upon Tyne NE1 7RU, UK; Matthew.Leach@newcastle.ac.uk

**Keywords:** horse, acute laminitis, Obel, Horse Grimace Scale, pain assessment

## Abstract

**Simple Summary:**

Acute laminitis is a common equine disease characterized by intense foot pain. This work aimed to investigate whether the Horse Grimace Scale (HGS), a facial-expression-based pain coding system, can be usefully applied to assess pain associated with acute laminitis in horses at rest. Ten horses, referred as acute laminitis cases with no prior treatment, were assessed at the admission and at seven days after the initial evaluation and treatment. The authors found that the Horse Grimace Scale is a potentially effective method to assess pain associated with acute laminitis in horses at rest, as horses showing high HGS scores also exhibited higher Obel scores, and veterinarians classified them in a more severe painful state.

**Abstract:**

Acute laminitis is a common equine disease characterized by intense foot pain, both acutely and chronically. The Obel grading system is the most widely accepted method for describing the severity of laminitis by equine practitioners, however this method requires movement (walk and trot) of the horse, causing further intense pain. The recently developed Horse Grimace Scale (HGS), a facial-expression-based pain coding system, may offer a more effective means of assessing the pain associated with acute laminitis. The aims of this study were: to investigate whether HGS can be usefully applied to assess pain associated with acute laminitis in horses at rest, and to examine if scoring HGS using videos produced similar results as those obtained from still images. Ten horses, referred as acute laminitis cases with no prior treatment, were included in the study. Each horse was assessed using the Obel and HGS (from images and videos) scales: at the admission (before any treatment) and at seven days after the initial evaluation and treatment. The results of this study suggest that HGS is a potentially effective method to assess pain associated with acute laminitis in horses at rest, as horses showing high HGS scores also exhibited higher Obel scores and veterinarians classified them in a more severe painful state. Furthermore, the inter-observer reliability of the HGS total score was good for both still images and video evaluation. There was no significant difference in HGS total scores between the still images and videos, suggesting that there is a possibility of applying the HGS in clinical practice, by observing the horse for a short time. However, further validation studies are needed prior to applying the HGS in a clinical setting.

## 1. Introduction

Acute laminitis is a common equine disease with an estimated prevalence worldwide of 7–14% [1,2]. In horses, laminitis can be an extremely painful condition, both acutely and chronically, with distinctive signs such as lameness, inability or reluctance to walk, frequent weight shifting, and abnormal weight distribution on hind feet to relieve the pressure on front feet. Despite acute laminitis being considered a global equine welfare problem [1,3], there is no gold standard for the quantification of pain caused by this disease and the evaluation of the efficacy of the pain relieving treatments routinely administered. The Obel grading system [4] is a simple descriptive scale developed to evaluate equine lameness; it is the most widely accepted method for describing the severity of laminitis by equine practitioners [5,6]. The clinical assessment of the Obel grade includes the observation of the horse walking and trotting in a straight line on a hard surface, away from and towards the assessor. The horse is then scored on a grading scale [7], ranging from 0 to 4, as described in Table 1. 

In the absence of a gold standard, the method currently preferred for describing a horse with laminitis is the Obel grading system, which categorises laminitis-associated lameness on the basis of the severity of the signs of pain. However, the Obel grade assessment has some limitations: firstly, it requires the horse to move (walk and trot) and this movement is likely to cause further pain, affecting its welfare. Secondly, the Obel grade has been shown to be only moderately reliable [5], with effectiveness being influenced by the experience of the assessor [6].

Therefore, there remains a requirement for a reliable and effective tool for the assessment of pain associated with laminitis in horses at rest. Recently, facial expressions have been investigated for pain assessment in several non-human mammal species, including horses [8,9,10,11,12]. The Horse Grimace Scale (HGS) is a facial-expression-based pain coding system and may potentially overcome the difficulties described with the Obel assessment [13]. Changes in the HGS have been shown to be detectable, without the need of approaching or moving the subject, by observers with only the HGS manual for guidance. HGS incorporates six Facial Action Units (FAUs) that are independently scored. These are: stiffly backwards ears, orbital tightening, tension above the eye area, prominent strained chewing muscles, mouth strained and pronounced chin, and strained nostrils and flattening of the profile. A detailed description and a graphic representation of individual action units are reported by Dalla Costa et al. [13]. Each FAU is scored from images on a 3-point scale, with zero indicating that the assessor is confident that the action unit is not present, one indicating that the assessor is confident that the action unit is only moderately present, and two indicating that the assessor is confident that the action unit is obviously present. The HGS has been shown to be a potentially valid measure of pain following routine castration surgery [13]. However, the authors concluded that the HGS should be further validated for other potentially painful conditions and its accuracy should be investigated in conditions more similar to the clinical context before it can be considered for clinical application [13]. Even though the use of images offers an appropriate means of experimentally validating the HGS for pain assessment, this does not reflect the clinical scenario where the veterinarian would want to simply observe the animal for a short time. 

The present study aims to investigate whether HGS can be usefully applied to assess pain associated with acute laminitis in horses at rest. Additionally, the authors aimed to examine if scoring HGS using videos produced similar results as those obtained from still images.

## 2. Materials and Methods

The study design was approved by the Brandenburg State Veterinary Authority (V3-2347-A-42-1-2012) in compliance with German legislation on animal experiments. Individual horse owner’s consent was obtained for all horses participating in this study. Horses involved in this study were admitted for routine veterinary treatment of acute laminitis at the request of their owner on a voluntary basis. Consequently, this was a purely observational study with no animals being treated for the purposes of this study. 

### 2.1. Animals

**Figure 1:**
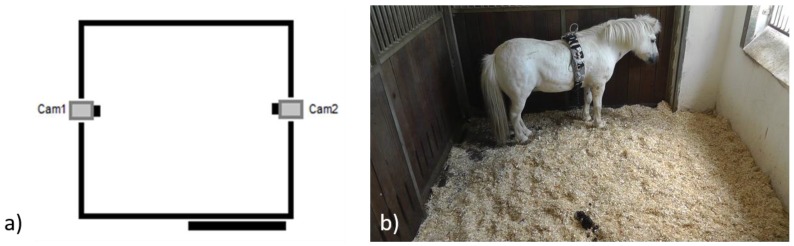
The drawing (**a**) shows the position of the two HD cameras; Picture on the right (**b**) shows a still image.

Ten horses of different breed, age, and gender (Table 2) were admitted to the Havelland Equine Clinic between 2012 and 2014. During this period, all referred horses that presented acute laminitis with no prior treatment were included. After admission, the subjects were stabled in standard single boxes (4 × 3 m with an outside window) on wood shavings (German Horse Span Classic, German Horse Pellets, Wismar Germany), and in visual contact with conspecifics. Two High Definition Cameras (Panasonic, HDC-SD99, Panasonic, Japan), were placed orthogonally on the top of the grate section on opposite sides of the box and allowed for video-recording of the behaviour and face of the horse without interfering with their normal behaviour (Figure 1).

For each horse, the routine treatment was: oral administration of Phenylbutazone (initial 4.5 mg/kg bwt every 12 h for two days and subsequent 2.5 mg/kg bwt every 12 h, Phenylbutariem^®^, Ecuphar, Germany); subcutaneous injections of heparin (50 IU/kg bwt every 12 h for five days, Heparin-Natrium-25000-ratiopharm^®^, Ratiopharm, Germany), padded hoof bandages with frog support and elevated heels (3–4 cm), ice water applications every 2 h for the first three days of treatment and strictly restricted movement in an individual box with a deep and soft bedding of wood shavings.

### 2.2. Obel Grade Scoring

One experienced veterinarian conducted the live evaluation of the lameness with the Obel scoring system. Each horse was assessed at admission to the clinic (before any treatment, TP1) and seven days after the initial evaluation and treatment (before the subject was discharged, TP7). All the assessments were carried out in the morning between 8 a.m. and 12 p.m. Each horse was evaluated while walking (20 m) and trotting (20 m) in a straight line on a firm surface, and scored according the definitions reported in Table 1. 

### 2.3. Horse Grimace Scale (HGS) Recordings

#### 2.3.1. Image Collection

For each horse, at TP1 and TP7, twenty-minute videos were simultaneously recorded using the two cameras placed on the top of the box. Following the methods of Dalla Costa and colleagues [13], still frames of the face of each horse were extracted from videos on every occasion they directly faced the video camera.

#### 2.3.2. Image Selection and Scoring

A non-participating treatment and time point blind assistant with no experience of assessing pain in horses randomly selected 40 still images (image set). In order to maintain a balanced design for the statistical analysis, the image set comprised two images of each horse at each time point, for a total of four images per horse. Images were organized in a random order using the software Random.org (https://www.random.org/sequences/). Four trained treatment and time point blind veterinarians (not involved in either the Obel scoring or the image selection) evaluated each image using the Horse Grimace Scale (HGS) [13]. A detailed hand out with the description of the FAUs and the scoring system was distributed to the veterinarians in order to be used as training materials (Table 3). The veterinarians were also asked to evaluate the intensity of pain for each image based upon their own clinical experience: 0 = no pain, 1 = mild, 2 = moderate, 3 = severe.

#### 2.3.3. Video Processing and Scoring

In addition to the images, fifteen-second clips were cropped from the original videos so that only the head of the horse was visible. Two video-clips of each horse, one at each time point (TP1 and TP7), were produced. Consequently, 20 video clips were analysed further. Two trained treatment and time point blind veterinarians (involved in the still image HGS scoring) watched the videos and provided a HGS score for each of them using the HGS [13]. The training was the same as for the veterinarians involved in the still image HGS scoring. The veterinarians scored each action unit based on the 15-sec clip observation.

### 2.4. Statistical Data Analysis

Statistical analysis was performed using SPSS 23 (SPSS Inc., Chicago, IL, USA). Statistical significance was accepted at *p* ≤ 0.05. The HGS total score for each image was calculated and consisted of the sum of the scores across the six FAUs. The data were tested for normality and homogeneity of variance using the Kolmogorov-Smirnov and Levene test, respectively. As both Obel and HGS data were not normally distributed, non-parametric statistical tests were applied to assess whether they changed at different time points (Wilcoxon’s signed rank test). The reliability of the HGS scale was determined using the inter-class correlation coefficient (ICC) to compare the HGS total score and each of the FAUs across all the participants. Interpretation of the ICC was based on Altman [14] and Landis and Koch [15], with the following divisions: ‘‘very good’’ (0.81–1.0), ‘‘good’’ (0.61–0.80), ‘‘moderate’’ (0.41–0.60), ‘‘fair’’ (0.21–0.40), ‘‘poor’’ (0.20). A Wilcoxon’s signed rank test was used to determine if the intensity of pain ascribed by veterinarians changed from TP1 to TP7. Spearman’s rank correlation coefficients (rs) were calculated to investigate the relationship between the HGS images’ score and the Obel grade and to determine if there was a difference in HGS total scores assessed from images compared to the video sequences.

## 3. Results

### 3.1. Obel Grade

**Figure 2:**
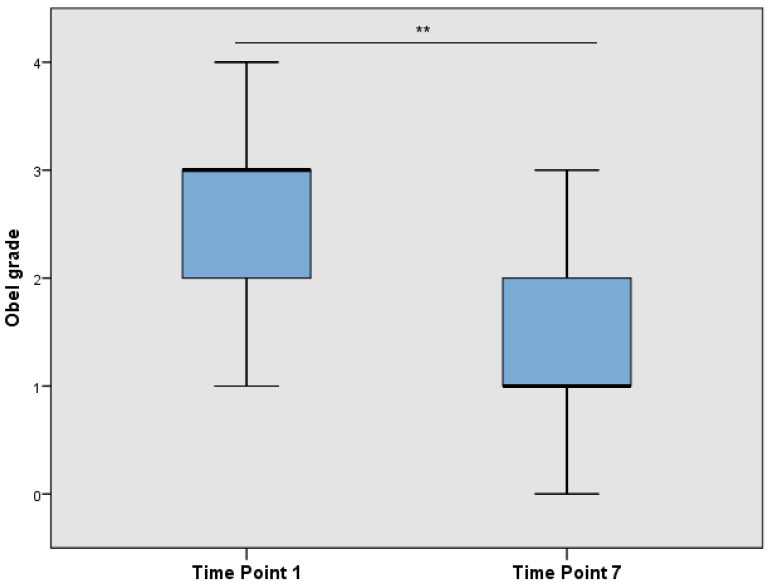
Obel grade over time drawn in a box plot (** *p* < 0.001).

At TP1 (after admission), the average Obel grade was 2.6 ± 1.0 (mean ± SD) and decreased significantly to 1.4 ± 0.8 (mean ± SD) (Wilcoxon’s signed rank test, *p* < 0.001) after the onset of treatment at TP7 (Figure 2). 

### 3.2. Scoring Images with HGS

**Figure 3:**
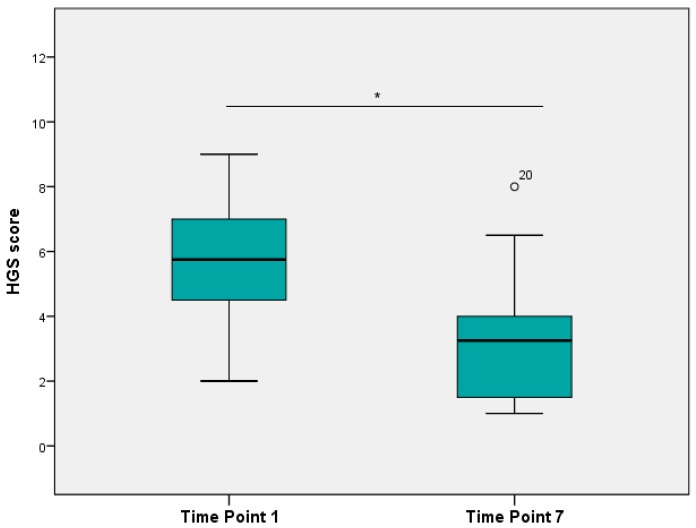
HGS scores from images by four treatment and time-point blind veterinarians over time drawn in a box plot (* *p* < 0.05). Outliers (1.5 to 3 times length of the box), labelled with the individual case numbers, are graphed as circles.

The average total HGS score from images at TP1 was 5.8 ± 2.0 (mean ± SD) and decreased significantly to 3.5 ± 2.3 (mean ± SD) at TP7 (Wilcoxon’s signed rank test, *p* < 0.01) (Figure 3).

The HGS demonstrated very good inter-observer reliability with an overall Interclass Correlation Coefficient (ICC) value of 0.85. The individual FAUs showed very good reliability with ICC values of: 0.95 for stiffly backwards ears, 0.93 for orbital tightening, 0.80 for mouth strained and pronounced chin. Good reliability was also demonstrated for strained nostrils and flattening of the profile (ICC = 0.76) and for tension above the eye area (ICC = 0.68). The only exception was for prominent strained chewing muscles, which presented only fair reliability (ICC = 0.44).

**Figure 4:**
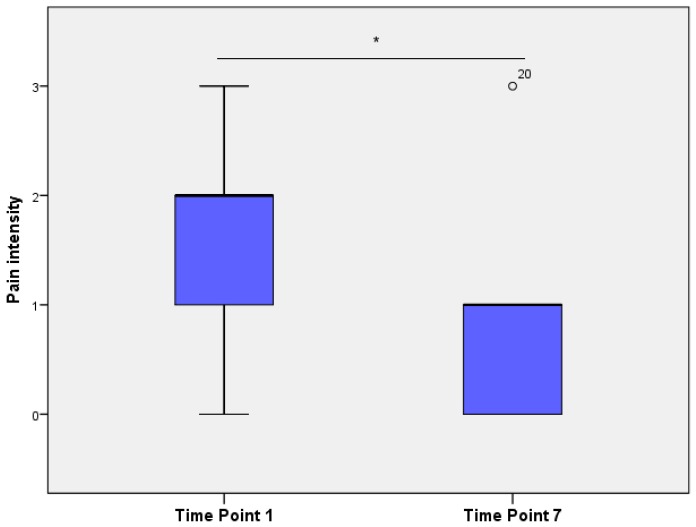
Pain intensity (descriptive pain scores), derived from images by four trained observers over time, drawn in a box plot (* *p* < 0.05). Outliers (1.5 to 3 times length of the box), labelled with the individual case numbers, are graphed as circles.

The average “pain intensity”, as evaluated by veterinarians, differed significantly between time points (Figure 4), being significantly higher in TP1 compared to TP7 (Wilcoxon’s signed rank test, *p* < 0.01).

### 3.3. Scoring Videos with HGS

The HGS score from videos at TP1 was 5.0 ± 2.6 (mean ± SD) and decreased to 3.4 ± 3.7 (mean ± SD) at TP7, although non-significantly. The HGS assessed on videos also showed a good inter-observer reliability with an overall ICC value of 0.74; most FAUs showed very good to fair inter-observer reliability: stiffly backwards ears (ICC = 0.92), prominent strained chewing muscles (ICC = 0.66), strained nostrils and flattening of the profile (ICC = 0.52), orbital tightening (ICC = 0.50), and mouth strained and pronounced chin (ICC = 0.40). Veterinarians poorly agreed on scores of tension above the eye area (ICC = 0.17). 

### 3.4. Relationship between Obel Grade and HGS

HGS total score assessed using images was correlated positively with the Obel grade (Spearman correlation, Rho = 0.653; *p* < 0.01) and with the pain intensity, evaluated by veterinarians (Spearman correlation, Rho = 0.873; *p* < 0.001).

### 3.5. Comparison of HGS Scores from Images and Video

There was no significant difference in HGS total scores between the still images and videos. Furthermore, HGS total score assessed using still images was positively correlated with HGS scored using videos (Spearman correlation, Rho = 0.607; *p* < 0.01).

## 4. Discussion

The results of this study suggest that HGS is a potentially effective method to assess pain associated with acute laminitis in horses at rest. In fact, veterinarians blinded to time and treatment condition of the patients, attributed significantly higher HGS scores to laminitic horses before the onset of treatment. Horses showing high HGS scores, also exhibited higher Obel scores and veterinarians classified them in a more severe painful state. 

The inter-observer reliability of the HGS total score was good for both image and video evaluation; these findings, similar to those of other grimace scales [9,10,11,13], confirm that a short training of assessors is sufficient to reliably apply this method. This is probably due to the fact that humans instinctively tend to focus on the head and face when assessing pain in people and other animals [16,17]. 

So far, the studies that have used facial expression for pain assessment in research settings, have used images captured from videos [12,13] as this step is essential in terms of developing and validating the method as a pain assessment tool [8]. However, images may not accurately reflect the potentially changing nature of facial expressions in real time [18] and to date they have always been scored at a later stage. In order to explore the possibility of applying the HGS in a clinical setting while maintaining a blinded-observer experimental design, short video-clips of the same horses were used, as already proposed in other species [18]. The results of this study show that there were no significant differences in HGS total scores between the scoring of still images and video sequences. However, the 15-second video clips seem more difficult to score for the observers as illustrated by the non-significant decrease in the HGS total score from TP1 to TP7, resulting from the high level of variation between the observers. The overall reliability was good, with the backwards ear position being the best action unit for both the still images and the videos. Prominent strained chewing muscles showed a better inter-observer reliability when scored from videos compared to still images; this was probably due to the fact that the persistent contraction of these muscles is more evident from videos. Two action units (i.e., mouth strained and pronounced chin, tension above the eye area) showed fair to poor inter-observer reliability, probably due to the fact that scoring videos poses different challenges compared to scoring still images with the expression of specific action units changing over time and complicating the assessment. In particular, 15-sec clips were reported by the assessors to be too short to integrate the information of facial movements in a judgement for each action unit. In order to solve this issue, observers could be asked to score longer video sequences (e.g., 1 min) after training with short video clips illustrating each action unit at each level on the scale (i.e., 0, 1, and 2). Short video sequences have already been utilised in the effective training of observers for other behavioural methods including pain specific behaviours (e.g., [19]) and Qualitative Behaviour Assessment (e.g., [20]). These methods rely on the ability of humans to integrate observed details of an animal’s behaviour [21]. 

Prior to applying live scoring of the HGS in a clinical setting, further validation studies are needed as scoring live (i.e., in a clinical context) does not appear to be as straightforward as scoring from images. This study represents a first step, as it compares the HGS scoring from still images to the HGS scoring from short video-clips. The influence of an observer on horse facial expressions needs to be further explored. In humans and other animal species, facial expressions of pain are already being used as a clinical tool for assessing acute pain in the presence of an observer (e.g., [18,22,23]). The possibility to assess pain in horses at rest using HGS would improve the welfare of horses with acute laminitis in several ways: the assessment itself is not painful for the subject and can be repeated several times. A valid and reliable assessment of pain is essential for effective pain management of horses with acute laminitis.

## 5. Conclusions

The HGS was determined to be a potentially effective method to assess pain associated with acute laminitis in horses at rest. However, further validation studies are needed prior to applying the HGS in a clinical setting, as scoring live does not appear to be as straightforward as scoring from still images. 

## Figures and Tables

**Table 1:** Obel grade according to Owens et al. [7].

Obel Grade	Description
0	No gait abnormalities
1	The horse exhibits a normal gait at a walk. The trot shows a shortened stride with an audible cadence abnormality, but shows even head and neck lifting for each foot.
2	The walk is stilted, but shows no abnormal head or neck lifting. The trot shows obvious lameness with uneven head and neck lifting. A forefoot can be lifted off the ground easily.
3	The lameness is obvious at a walk and trot. The horse resists attempts to have a forefoot lifted and is reluctant to move.
4	The horse experiences difficulty bearing weight at rest or is very reluctant to move.

**Table 2:** Breed, gender, and age of the horses recruited for the study.

Horse ID	Breed	Gender	Age
1	pony	mare	4
2	Welsh Cob D	gelding	8
3	Icelandic Horse	mare	4
4	Shetland Pony	gelding	13
5	pony	gelding	17
6	Haflinger	mare	15
7	pony	mare	15
8	Haflinger	mare	6
9	Icelandic Horse	mare	6
10	pony	gelding	6

**Table 3:** Description of each Facial Action Unit (FAU) of the Horse Grimace Scale (HGS) as reported in the handout distributed to the veterinarians.

Facial Action Unit (FAU)	Description
Stiffly backwards ears	The ears are held stiffly and turned backwards; movements are limited also in presence of environmental stimuli
Orbital tightening	The eyelid is half-closed or closed, the orbit is contracted, eyes are not focused on the environment
Tension above the eye area	Increased muscle tension in the area above the eyes, the underlying bone structure becomes clearly visible
Prominent strained chewing muscles	Increased tension of the chewing muscles, that becomes prominent and clearly recognizable
Mouth strained and pronounced chin	Strained mouth, the corner of the lips is shortened, the lower lip is tense, the chin is contract and becomes more pronounced (crescent-shaped)
Strained nostrils and flattening of the profile	The nostrils are dilated and strained, the profile changes and you can see two bulges (one at the nostrils and upper lip)
